# Pharmacokinetics of pradofloxacin, florfenicol, and tulathromycin and response to treatment of steers experimentally infected with *Mannheimia hemolytica*

**DOI:** 10.1111/jvim.17270

**Published:** 2024-12-10

**Authors:** Derek M. Foster, Jennifer L. Halleran, Megan E. Jacob, Stephanie Hempstead, Luke B. Borst, Tatiane T. Negrao Watanabe, Hiroko Enomoto, Mark G. Papich

**Affiliations:** ^1^ Department of Population Health and Pathobiology, College of Veterinary Medicine North Carolina State University Raleigh North Carolina USA; ^2^ Antech Diagnostics, Inc. Phoenix Arizona USA; ^3^ Department of Molecular and Biomedical Sciences, College of Veterinary Medicine North Carolina State University Raleigh North Carolina USA

**Keywords:** antibiotics, bovine respiratory disease, fluoroquinolone

## Abstract

**Background:**

Bovine respiratory disease (BRD) is an economically important disease in the beef industry, and a major driver of therapeutic antibiotic use. Pharmacokinetic data of these drugs is relatively limited in diseased animals.

**Hypothesis/Objective:**

To determine the concentrations of pradofloxacin, florfenicol, and tulathromycin in the airways, plasma, and interstitial fluid (ISF) of steers with a clinically relevant model of bacterial respiratory disease.

**Animals:**

Twenty‐four Holstein and Holstein/Jersey cross steers ranging in age from 6 to 15 months.

**Methods:**

A randomized, blinded clinical trial was performed. After transport stress, steers were inoculated with *Mannheimia hemolytica* to induce BRD. Upon onset of clinical disease, steers were treated with pradofloxacin, florfenicol or tulathromycin. Blood, ISF, and pulmonary epithelial lining fluid (PELF) samples were obtained for drug concentration determination. Clinical exams and thoracic ultrasound examinations were conducted daily. Animals were euthanized at the end of the study period to assess lung lesions.

**Results:**

Pradofloxacin *C*_max_ in PELF was 0.81 μg/mL (CV = 49.02%) and penetration into the PELF was 203.58% (72%). Florfenicol *C*_max_ in PELF was 2.94 μg/mL (42.1%) and penetration was 230.08% (78.82%). Tulathromycin PELF *C*_max_ was 0.9 μg/mL (45.03%) and PELF penetration was 518.97% (56.59%).

**Conclusions and Clinical Importance:**

There are differences in penetration of the drugs into the ISF and PELF compared to one another and previous data from healthy steers demonstrating the effect of disease on the PK of these drugs.

**Abbreviations**
AUC: area under the concentration
BALF: broncho alveolar lavage fluid
BRD: bovine respiratory disease
*C*_max_
: maximum drug concentration
FLOR: florfenicol/flunixin meglumine
HPLC: high‐pressure liquid chromatography
ISF: interstitial fluid
LOD: limit of detection
LOQ: limit of quantification
MALDI‐TOF: matrix assisted laser desorption ionization time of flight
MH: *Mannheimia hemolytica*
MHB: Mueller‐Hinton broth
MIC: minimum inhibitory concentrations
MS/MS: tandem mass spectrometric
NP: nasopharyngeal
PELF: pulmonary epithelial lining fluid
PFGE: pulsed‐field gel electrophoresis
PK: pharmacokinetics
PK/PD: pharmacokinetic‐pharmacodynamic
PRA: pradofloxacin
QC: quality control
*T*_MAX_
: time to peak concentration
TUL: tulathromycin
UPGMA: unweighted‐pair group method
UPLC: ultra‐performance liquid chromatography
UV: ultraviolet

## 1 INTRODUCTION

Bovine respiratory disease (BRD), the most important health challenge in the beef industry,^1^ is a multifactorial disease leading to lower respiratory bacterial infections.^2^ *Mannheimia hemolytica* (MH) is the most common bacterial pathogen in beef cattle with BRD.^3^ Fluoroquinolones are commonly used for treatment of BRD because of their gram‐negative spectrum, bactericidal activity, airway penetration, and efficacy.^1^, ^4^, ^5^, ^6^, ^7^ Fluoroquinolone resistance among BRD pathogens is increasing.^8^, ^9^, ^10^ Pradofloxacin, a third generation fluoroquinolone, is approved for use in companion animals in the United States and recently approved for use in cattle and swine.

Dosing regimens are based on plasma pharmacokinetics (PK) or tissue homogenates, which are poor predictors of drug concentrations in the airways.^5^, ^11^, ^12^, ^13^, ^14^, ^15^ More recent research has demonstrated the possibility of directly sampling the lower airway by collecting pulmonary epithelial lining fluid (PELF) via a guarded swab or bronchoalveolar lavage fluid (BALF). The BALF method samples a larger area of the lung, but BALF samples can be contaminated with leukocytes from the airways, which can contain high antibiotic concentrations. The BALF sample is diluted from infusion of saline, adding variability in the measurement.^16^ Collection of PELF using a guarded swab samples a smaller area of the lung, but the drug can be directly measured from fluid extracted from the swab, minimizing the variability because of collection and maximizing sampling frequency.^5^

In BRD, there is infiltration of inflammatory exudates into the airway and disruption of the blood‐alveolar barrier. Effusions into the airways may dilute the drug, or alternatively increase drug concentrations because of high drug protein binding or concentration of the drug in leukocytes. The few studies comparing PK of antibiotics in cattle with BRD have demonstrated differences that could change the prediction of success.^17^, ^18^

The objective of this study was to determine the concentrations of pradofloxacin, florfenicol, and tulathromycin in the airways, plasma, and interstitial fluid (ISF) of steers with a clinically relevant BRD model.

## 2 MATERIALS AND METHODS

### 2.1 Animals

Twenty‐four Holstein and Holstein/Jersey cross steers ranging in age from 6 to 15 months with a mean body weight of 238.73 kg (range, 87‐390 kg) were obtained. All animals were in good health based upon initial physical examination and had no known antimicrobial treatments 14 days before enrollment. This study was approved by the NC State University Institutional Animal Care and Use Committee.

### 2.2 Allocation of animals

Before enrollment, animals were randomly assigned into groups of 3 and then subsequently assigned to a treatment group, known only to the un‐blinded study coordinator, who was not involved in health scoring, thoracic ultrasonography, or lung scoring. Randomization was achieved through assignment of steers in numerical order to pre‐designed forms generated by the sponsor. The order in which the treatment groups were prefilled was based upon generating random numbers between 0 and 1 for each possible animal (SAS 9.4; Cary, North Carolina). The 3 treatments were: pradofloxacin (Pradalex, 200 mg/mL; Elanco Animal Health, Greenfield, Indiana; PRA), administered at 10 mg/kg subcutaneously in the neck; tulathromycin (Draxxin, 100 mg/mL, Zoetis, Inc, Parsippany‐Troy Hills, New Jersey; TUL) administered at 2.5 mg/kg, subcutaneously in the neck; florfenicol/flunixin meglumine (Resflor Gold, 300 mg/mL, 16.6 mg/mL, Merck Animal Health, Inc., Rahway, New Jersey; FLOR) administered at 40 mg/kg; 2.2 mg/kg subcutaneously in the neck. These treatments were administered by the study coordinator after disease onset.

### 2.3 Disease induction model

An isolate of MH from a clinical BRD sample was obtained from the Wisconsin Veterinary Diagnostic Laboratory and frozen in Tryptic Soy Broth with 25% glycerol at −80°C before use. A day before inoculation, the isolate was streaked onto Tryptic Soy Agar plates with 5% sheep blood (BAP; Remel Columbia Blood Agar w/3% Sheep Blood, Remel, Lenexa, Kansas) and incubated at 37°C for 18 to 22 hours. After incubation, isolated colonies were suspended in 100 mL of Luria Broth (LB) in triplicate and incubated in a shaker at 37°C for at least 5.5 hours. The suspension was centrifuged at 2000*g* for 5 minutes. The supernatant was removed, and the pellet was washed 3 times in 10 mL of sterile phosphate‐buffered saline (PBS; ThermoFisher Scientific, Waltham, Massachusetts). The pellet was suspended in 50 mL of sterile PBS and adjusted to a final concentration of 1 × 10^9^ cfu/mL. The final sample was distributed into 40 mL sterile aliquots. The starting bacterial concentration was confirmed for each aliquot by serially diluting and spot‐plating 20 μL of each dilution onto a BAP in triplicate, incubating plates overnight at 37°C, and counting the colonies on the appropriate dilution.

Animals were transported approximately 900 km, starting at their farm of origin with an overnight stay at a commercial sale barn. Upon arrival at the research facility, the steers were housed individually in a barn maintained between 21°C and 24°C. Animals were weighed and examined on arrival. Jugular catheters and subcutaneous ultrafiltration probes were placed at that time. Then, each animal was experimentally infected with MH as described before with slight modifications.^19^ The animals were restrained in a head catch and a mask (Equi‐Resp, Blanchard, Oklahoma) attached to an ultrasonic nebulizer (Salter AIRE Elite Compressor, Salter Labs, Arvin, California) was placed over the nostrils of the animal. They were nebulized with 5 mL of 1 × 10^9^ cfu/mL of MH. Administration continued until the entire contents of the nebulizer reservoir was delivered to the animal (mean time of nebulization = 10.81 minutes). After nebulization, 35 mL of 1 × 10^9^ cfu/mL of MH was instilled into the lower airways via nasotracheal intubation through a broncho‐alveolar lavage catheter wedged into a bronchus.

### 2.4 Clinical disease monitoring

Animals were examined every 12 hours after disease induction by a single blinded observer until they met criteria for treatment and then every 24 hours until euthanasia. Rectal temperature, depression score (Table 1), and respiratory score (Table 2) were assessed at each of these examinations by this same blinded observer.^20^ Animals were determined to meet inclusion criteria for treatment once they met 2 of the 3 following criteria; depression score ≥2, respiratory score ≥1, and/or rectal temperature ≥39.5°C. Thoracic ultrasonography was performed by the same blinded clinician (DF) at each health observation as previously described using a 6.2 MHz linear probe (Ibex EVO, EI Medical Imaging, Loveland, Colorado).^21^ Areas of consolidation greater than 1 cm^2^ were considered lesions.^22^, ^23^

**TABLE 1:** Depression score.

0 = Normal: Alert, active, normal appetite, well‐hydrated, coat normal
1 = Mild Depression: Moves slower than normal, slightly rough coat, may appear lethargic, but upon stimulation appears normal
2 = Moderate Depression: Inactive, may be recumbent, but is able to stand, gaunt, may be dehydrated
3 = Severe Depression: Down or reluctant to get up, gauntness evident, dehydrated
4 = Moribund: Unable to stand, approaching death; highly unlikely to respond to any antimicrobial therapy

**TABLE 2:** Respiratory score.

0 = Normal: No abnormal respiratory clinical signs. Respiratory rate and effort are appropriate for the environment
1 = Mild Respiratory Distress: Serous nasal or ocular discharge and/or cough
2 = Moderate Respiratory Distress: Mucous or mucopurulent nasal or ocular discharge and/or increase in respiratory rate or effort
3 = Severe Respiratory Distress: Marked increase in respiratory rate or effort, with 1 or more of the following: open mouth breathing, abdominal breathing and/or extended head.

### 2.5 Drug sample collection

**FIGURE 1:**
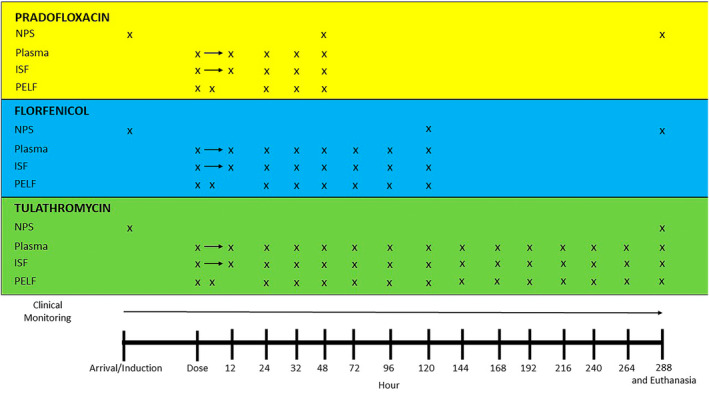
Summary of sampling protocol for each group. Each X indicates a sample collection timepoint for each group. The arrows indicate times in which multiple samples were taken. ISF, interstitial fluid; NPS, nasopharyngeal swab; PELF, pulmonary epithelial lining fluid.

Blood samples were collected via a catheter (MILACATH Extended Use 14Ga × 13 cm, MILA International, Inc., Florence, Kentucky) placed in the jugular vein as previously described.^5^ An ultrafiltration probe (RUF‐3‐12, BASi, West Lafayette, Indiana) was inserted in the subcutaneous space above the withers and ISF was collected as previously described.^5^, ^24^ Pulmonary epithelial lining fluid was collected as previously described.^5^ Figure 1 summarizes the sampling of steers in each group.

### 2.6 Bacterial sample collection and necropsy

Before experimental infection, a deep nasopharyngeal swab (Double Guarded Culture Swab, Jorgensen Labs, Loveland, Colorado) was collected for aerobic bacterial culture. Similarly, swabs were taken on the last day of blood sampling (48 hours for PRA, 120 hours for FLOR, and 288 hours for TUL) and immediately before euthanasia. Two weeks after the experimental infection, all animals were humanely euthanized (Euthasol, Virbac AH, Inc., Fort Worth, Texas), and a necropsy was performed immediately. Consolidation was estimated to the nearest 5% of each lung lobe.^25^ Samples were collected from lung tissue of all animals and additional lung tissue and swab samples were taken where visible lung lesions were present. Tissue and swab samples were immediately submitted for bacterial culture.

### 2.7 Bacterial culture

Nasopharyngeal swabs were submitted for aerobic bacterial culture. At necropsy, lung tissue was collected, dipped in 95% ethanol and flamed to decontaminate the surface from the collection process. The tissue was placed in a Whirl‐Pak filter bag with 1 mL of thioglycollate broth (BD, Franklin Lakes, New Jersey) and ground with a rubber roller. The resulting homogenate was then plated onto chocolate agar (Hardy Diagnostics, Santa Maria, California), BAP, MacConkey agar (Remel Thermo Scientific, Waltham, Massachusetts), and enriched in 9 mL of thioglycollate broth. Swabs of consolidated lung tissue were collected at necropsy and plated as above. Chocolate and BAP plates were incubated in 5% CO_2_ at 37°C. MacConkey agar and thioglycollate broths were incubated at 37°C in an ambient air incubator. Plates were incubated for up to 48 hours for evaluation of bacterial growth. Any isolate that was obtained from any sample type was struck for isolation and identified using matrix assisted laser desorption ionization (MALDI‐TOF; VITEK‐MS, bioMerieux, Marcy‐l Etoile, France).

### 2.8 Minimal inhibitory concentrations

Minimal inhibitory concentrations (MIC) were determined for all MH isolates before inoculation and those identified from tissue and swab samples using broth microdilution according to the Clinical Laboratory Standards Institute.^26^ Isolates from animals treated with PRA were streaked individually on a BAP and incubated at 37°C for 18 to 22 hours. Pradofloxacin powder was dissolved in sterile water and diluted in LB for a concentration of 32 μg/mL, and then serially diluted in the wells of a 96‐well plate from 16 to 0.015 μg/mL in LB broth. One to 2 bacterial colonies were suspended in sterile PBS and adjusted to 0.5 McFarland standard. Five microliters of suspension were added to each well for each isolate, as well as positive control wells; negative control wells contained only LB broth. The plates were incubated at 37°C for 24 hours and evaluated for visible growth of bacteria. MICs were established for isolates for florfenicol and tulathromycin using commercially available Sensititre Bovine/Porcine BOPO6F Vet AST Plates. Colonies were streaked as above and suspended into Mueller Hinton Broth. The suspensions were inoculated onto BOPO plates, incubated at 37°C for 18 hours, and read and interpreted automatically using a TREK ARIS 2X with Optiread.

### 2.9 Pulse field gel electrophoresis

All MH isolates were evaluated for relatedness using pulsed‐field gel electrophoresis (PFGE).^27^ MH isolates were streaked onto BAP and incubated at 37°C for 18 hours. Colonies were suspended in a 100 mM Tris and 100 mM EDTA buffer (pH = 8.0), and the concentration was adjusted to an optical density (OD) of 0.48‐0.52 (Dade Behring MicroScan Turbidity Meter; Siemens Medical Solutions, Malvern, Pennsylvania). The adjusted cell suspension was lysed with proteinase K (20 mg/mL) (Roche, Indianapolis, Indiana), and intact genomic DNA was digested with *SmaI* restriction enzyme (50 U) (Roche) in agarose‐embedded plugs.^28^ DNA fragments were separated via electrophoresis in 0.5× Tris‐Borate‐EDTA Buffer (TBE; BioRad, Hercules, California), 1% pure agarose (Seakam Gold Agarose, Lonza, Maine) for 18 hours at 14°C in a PFGE (CHEFF DR III, BioRad, Hercules, California) using pulse times of 2.2‐63.8 seconds. The reference strain *S*. Braenderup *H9812* was included as a marker and digested with *Xba*I. Gels were stained with ethidium bromide (10 mg/mL) (Sigma, St Louis, Missouri) for 30 minutes in 400 mL of reagent grade water. The gels were washed and photographed under UV light. PFGE images were analyzed using BioNumerics software version 7.1 (Applied Maths, Belgium).

### 2.10 Pradofloxacin plasma protein binding

In vitro plasma protein binding was determined by methods developed in our laboratory^5^ using an in vitro microcentrifugation system (Centrifree Micropartition system, Amicon Millipore, Billerica, Massachusetts) at 3 concentrations: 0.1, 1, and 10 μg/mL.

### 2.11 Drug analysis

Plasma, ISF, and PELF samples were analyzed for pradofloxacin by reverse phase high‐pressure liquid chromatography (HPLC) with ultraviolet (UV) detection, based on previous fluoroquinolone analysis.^24^ Plasma samples were extracted using solid phase extraction (Waters Corporation, Milford, Massachusetts). ISF was analyzed directly with no extraction. For pradofloxacin in PELF samples, 1 mL of a solution of 85% buffer solution and 15% methanol was added to the vial containing the filter paper used for collection. This was vortexed and incubated for 10 minutes at room temperature. After incubation, the vial was centrifuged for 10 minutes, and the clear supernatant was used for HPLC injection. The final concentration reported was adjusted for the volume used for extraction, the volume of fluid collected in the filter paper, and the measured recovery of drug from the filter paper. Analytical separation was accomplished with reverse‐phase HPLC (Agilent Technologies, Wilmington, Delaware). All samples were analyzed with UV detection at a wavelength of 279 nm, with a mobile phase of 75% buffer and 25% acetonitrile at a flow rate of 1 mL/min. The concentration of 0.1 μg/mL was used as the lower limit of quantification (LOQ) for plasma based on signal/noise ratio. For the ISF, the calibration curve was extended to 0.01 μg/mL, with acceptable LOQ criteria. The calibration curve for PELF samples consisted of the drug reference standard diluted in the extraction solvent to construct a calibration curve of 6 standards ranging from 10 to 0.01 μg/mL.

The samples were analyzed for florfenicol using reverse phase HPLC with UV absorbance detection for plasma samples and fluorescence detection for tissue fluids with methods previously used and validated in our laboratory,^5^ which had been modified from others.^29^, ^30^ Analysis of tulathromycin in plasma, ISF and PELF were performed by ultra‐performance liquid chromatography (UPLC) and tandem mass spectrometric (MS/MS) detection (Waters Corporation, Milford, Massachusetts) as previously described.^5^, ^18^

### 2.12 Pharmacokinetic analysis and statistical analysis

The pharmacokinetic analysis was conducted utilizing Phoenix WinNonlin (V. 8.4. Certara, St. Louis, Missouri). Drug concentrations of each drug for each animal were subject to noncompartmental analysis to estimate various pharmacokinetic parameters as previously described.^5^ Penetration factor was calculated from the AUC ratios of each drug in the plasma to either ISF or PELF as described.^24^

Because of the inability to statistically evaluate non‐parametric clinical scores over time, the respiratory and depression scores were visually assessed to determine clinical importance. To compare the percentage of calves with ultrasound lesions over time, a one‐way repeated measures ANOVA was used with a post‐hoc Bonferroni *t*‐test. To assess if there was a statistically significant difference between treatment groups and growth of MH at end of treatment and necropsy, a Kruskal‐Wallis ranked sum test was conducted. To determine if there was a statistically significant difference in percent lung consolidation between treatment groups, the amount of consolidation found at necropsy was initially summed from each individual lung lobe, and a Kruskal‐Wallis test was used.

## 3 RESULTS

### 3.1 Pharmacokinetic parameter estimates

**FIGURE 2:**
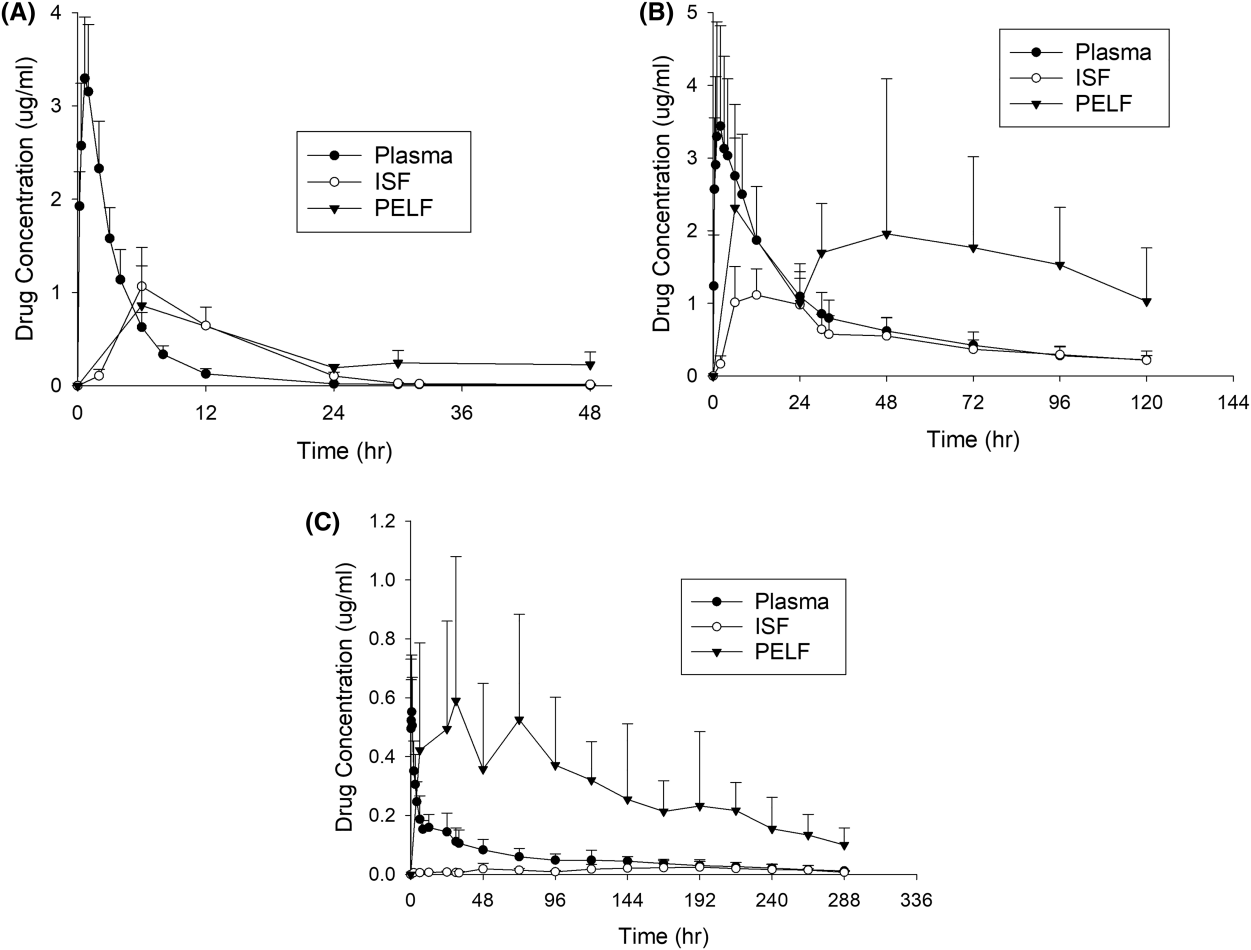
Drug concentrations for pradofloxacin (A), florfenicol (B), and tulathromycin (C) in plasma, interstitial fluid (ISF), and pulmonary epithelial lining fluid (PELF) in 8 steers experimentally infected with *Mannheimia hemolytica*.

Drug concentrations in each matrix for each drug are presented in Figure 2, and the pharmacokinetic data are presented in Table 3.

**TABLE 3:** Pharmacokinetics of pradofloxacin (10 mg/kg SC), florfenicol (40 mg/kg SC), and tulathromycin (2.5 mg/kg SC) in 8 calves with induced respiratory disease.

Parameter	Unit	Plasma		ISF		PELF	
		Geometric mean	Geometric CV%	Geometric mean	Geometric CV%	Geometric mean	Geometric CV%
Pradofloxacin							
*T*_max_	h	0.74	18.70	1.57	297.76	7.34	61.83
*C*_max_	μg/mL	3.40	18.17	0.73	192.65	0.81	49.02
AUC	h × μg/mL	13.19	21.72	6.39	113.72	27.53	55.30
Half‐life	h	4.79	26.52	7.88	45.06	24.94	176.98
Penetration	%			48.49	113.13	203.58	72.00
Florfenicol							
*T*_max_	h	1.54	78.28	4.84	273.47	20.48	219.04
*C*_max_	μg/mL	3.70	30.19	1.17	37.15	2.94	42.10
AUC	h × μg/mL	101.96	31.47	66.74	32.48	234.60	54.37
Half‐life	h	37.29	28.86	53.12	36.05	35.86	88.34
Penetration	%			65.46	14.58	230.08	78.82
Tulathromycin							
*T*_max_	h	0.46	52.72	63.05	82.52	33.57	108.53
*C*_max_	μg/mL	0.61	34.45	0.02	78.16	0.90	45.03
AUC	h × μg/mL	16.59	37.99	11.67	104.55	91.91	33.75
Half‐life	h	63.67	27.58	290.32	189.90	62.68	92.38
Penetration	%			21.12	65.86	518.97	56.59

Abbreviations: AUC, area under the curve from time 0 to infinity; *C*_max_, peak concentration; Penetration, the percent of AUC derived from a ratio of tissue fluid/plasma; *T*_MAX_, time to peak concentration.

For PRA, the peak concentration was attained rapidly with a plasma *T*_MAX_ of less than 1 hour. The concentrations in the ISF equilibrated with the plasma quickly, and PELF concentrations met or exceeded the plasma concentrations within 6 hours (Figure 2A). The mean elimination half‐life was approximately 5 hours in the plasma, 8 hours in the ISF, and almost 25 hours in the PELF.

The penetration into the ISF was 48% (Table 3), which closely reflects the fraction unbound as predicted by the in vitro protein binding assay (Table 4). The penetration into the PELF exceeds the ISF concentrations with greater than 200% penetration. The AUC was 9.63 h μg/mL in the PELF, demonstrating higher drug exposure at the site of infection compared to the ISF or plasma. The MH isolate used had an MIC of 0.015 μg/mL for pradofloxacin, and the mean PELF concentrations exceeded this value at all measured time points (Figure 2A).

**TABLE 4:** Pradofloxacin protein binding.

Concentration	10 mg/L	1 mg/L	0.1 mg/L
Mean % binding	45.75	41.76	44.08
Std. Dev.	0.97	1.41	3.96
Fraction unbound	0.54	0.58	0.56

FLOR demonstrated a rapid *T*_MAX_ with equilibrium between plasma and ISF. FLOR quickly diffused into the PELF with high concentrations observed at the first sampling time of 6 hours (Figure 2B). The penetration into the ISF was 65%, and this was exceeded by the penetration into the PELF (230%; Table 3). The PELF concentrations exceeded the MIC for FLOR of the MH isolate used in the study (0.5 μg/mL) at all measured time points (Figure 2B).

TUL demonstrated a short *T*_MAX_ and rapid equilibrium with the PELF that greatly exceeded the plasma or ISF concentrations (Figure 2C). The penetration into the ISF was 20%; whereas the penetration into the PELF was 520% (Table 3). TUL mean concentrations in the PELF did not exceed the MIC of the MH isolate (8 μg/mL) at any time point in the study.

### 3.2 Clinical outcomes

**FIGURE 3:**
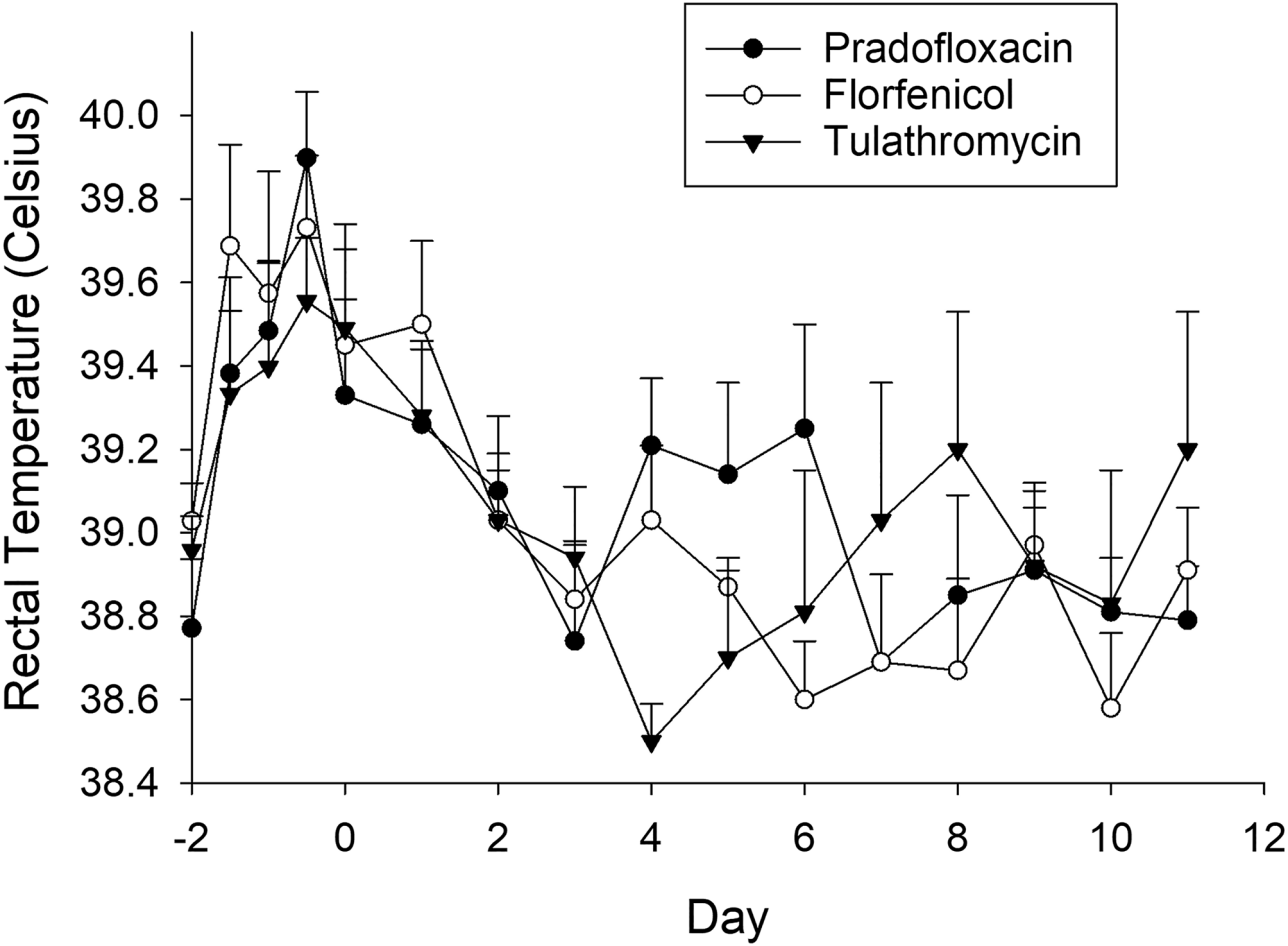
Clinical assessment of steers. The mean and standard error of rectal temperature of steers in each treatment group over time. Day 0 indicates time of treatment. There were no statistically significant differences seen between treatment groups.

**FIGURE 4:**
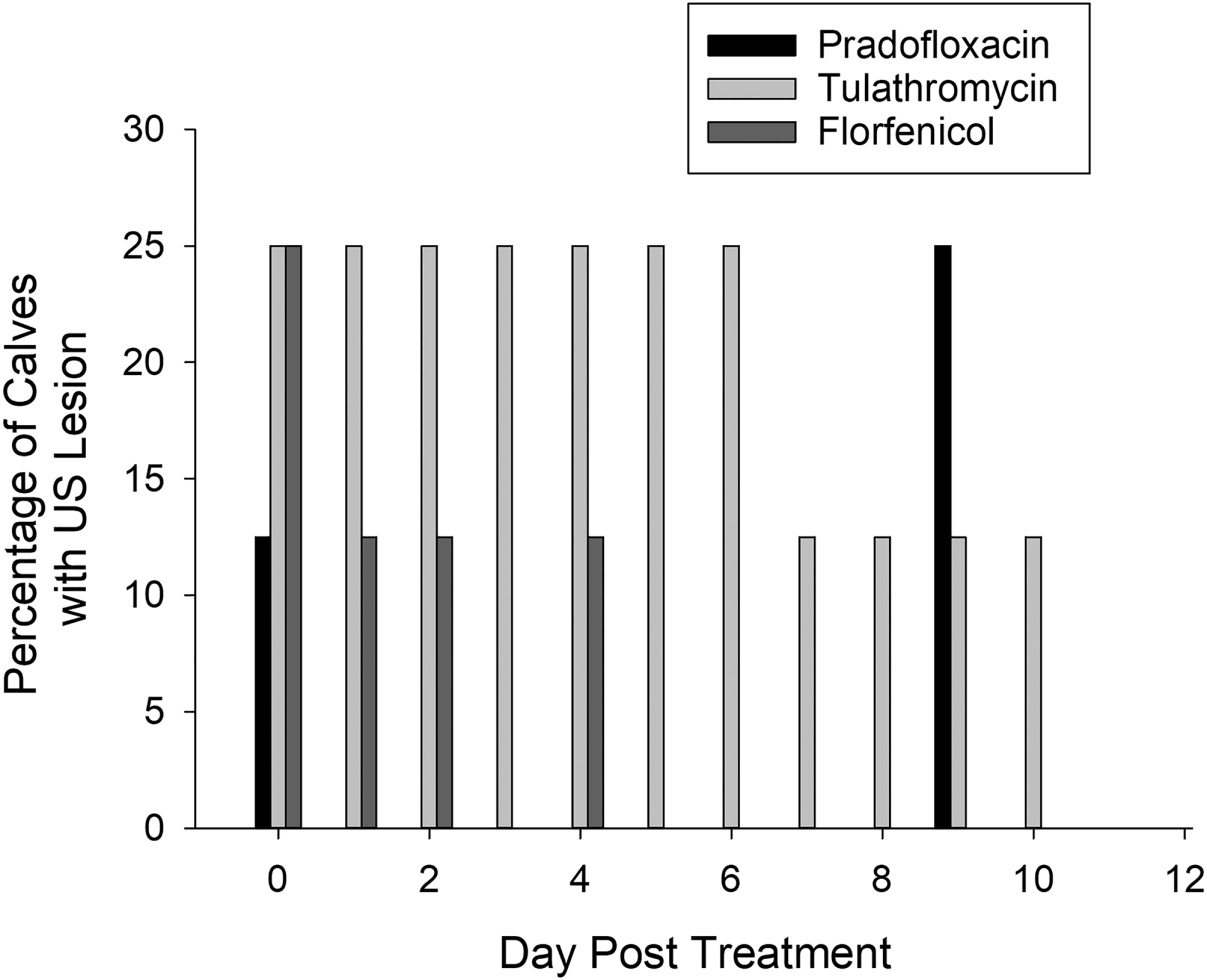
Percentage of calves with ultrasonographic lung lesions. Percentage of calves with lung consolidation greater than 1 cm^2^ on ultrasound over time for each treatment group. The percentage of calves with lesions was significantly greater (*P* < .001) in the TUL group compared to either PRA or FLOR.

All animals were healthy on arrival with depression and respiratory scores of zero and rectal temperatures less than 39.5°C. No lesions were observed during thoracic ultrasound on arrival. After treatment, steers in all 3 groups improved rapidly with no clinically relevant differences observed between the groups in depression score, respiratory score, or rectal temperature (Figure 3). The percentage of calves with ultrasonographic lung lesions was higher over time in the TUL group (*P* < .001) than either the FLOR or PRA groups (Figure 4).

### 3.3 Culture results and necropsy findings

**FIGURE 5:**
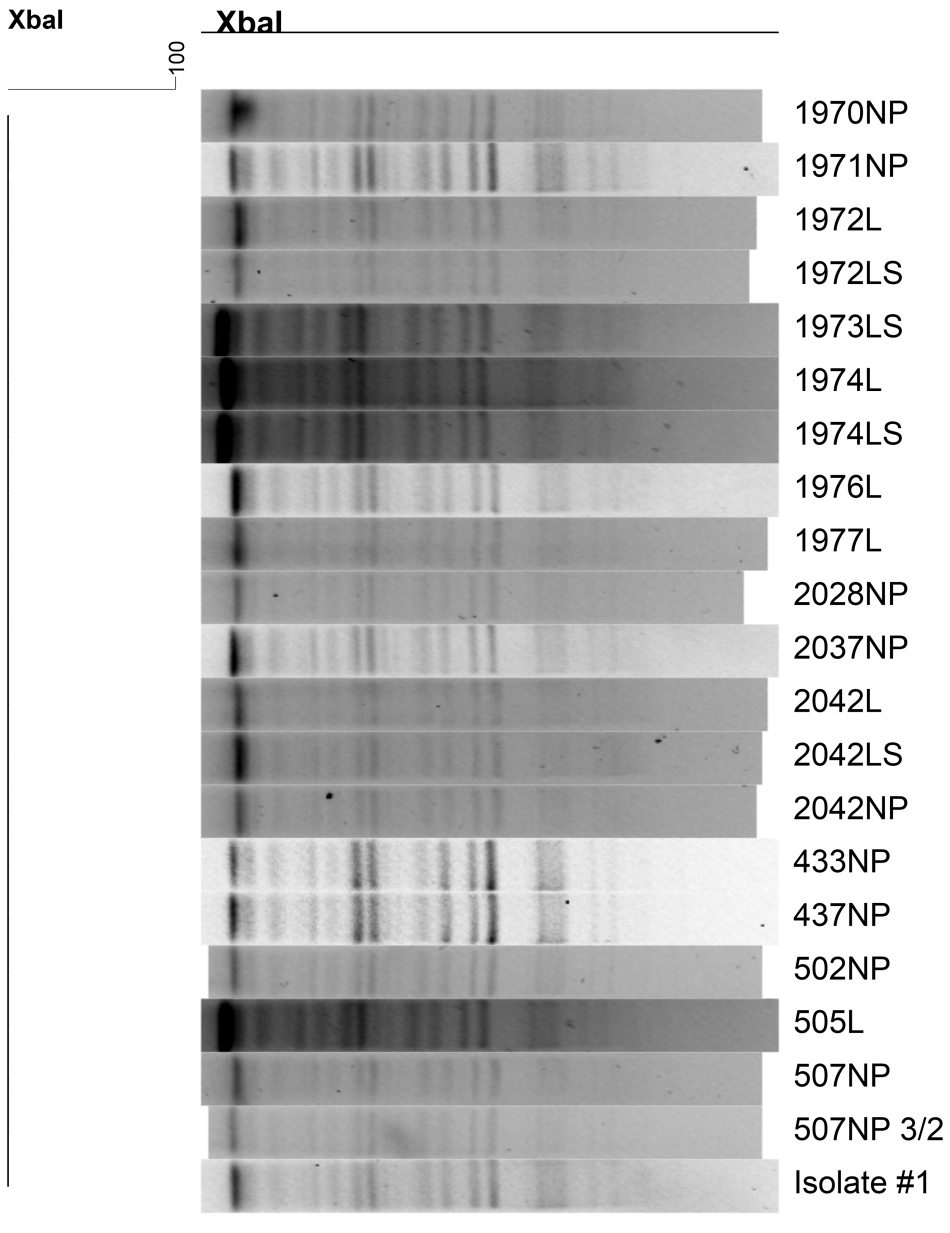
PFGE pattern of *Mannheimia hemolytica* isolated from steers by nasopharyngeal swab (NP), lung tissue (L), or lung swab (LS). The original isolate is indicated by “Isolate #1.”

Twenty‐one MH isolates were recovered from 15 steers. One FLOR steer had MH isolated on arrival, but was not positive for MH at any subsequent sampling. Seven isolates were found after treatment in 6 PRA steers. Nine isolates were obtained after treatment from 6 FLOR steers. There were 4 MH isolates from 3 TUL steers after treatment. There was no significant difference detected between groups in the number of steers with MH detected at the end of treatment (*P* = .46) or at necropsy (*P* = .52). All isolates had an MIC value that was the same or within 1 dilution from the original isolate. PFGE showed all isolates had the same banding pattern as the original isolate (Figure 5). At necropsy, 3 calves in each group had no areas of consolidation found. In calves with consolidation, this was exclusively found on the right side most commonly in the diaphragmatic lobe. Percentage of lung consolidation was 7.7 ± 3.4% for PRA, 8.6 ± 2.7% for FLOR, and 8.8 + 3.4% for TUL. These differences in consolidation were not statistically significantly different.

## 4 DISCUSSION

This work expands upon previous work to understand antibiotic PK in airways of cattle with respiratory disease.^17^, ^18^ Pradofloxacin was studied because it was recently approved for use in cattle. This study's experimental design and methods were similar to a previous study conducted in healthy cattle, allowing comparison of results for both tulathromycin and florfenicol in the diseased state,^5^ furthering our understanding of the impact of BRD on the PK of these antibiotics.

The PELF antibiotic concentration is representative of the extracellular environment of pulmonary pathogens.^31^, ^32^ In comparison to enrofloxacin in healthy steers,^5^ pradofloxacin attained an earlier *T*_max_ in the plasma (0.74 vs 4.65 h) and ISF (1.57 vs 15.6 h), and similar *T*_max_ in PELF (7.34 vs 6.0 h). Whether these differences are because of disease, drug differences, or variation between studies is undetermined. The unbound fraction of pradofloxacin was approximately 55% (Table 4) which approximates the penetration of unbound drug into the ISF (45%). Penetration into the PELF was over 200%, which is higher than enrofloxacin in healthy steers (24%).^5^ Factors that could produce higher concentrations in PELF for pradofloxacin than expected include active drug transport or the drug concentrating in leukocytes.^33^ Alternatively, inflammation in the lungs could increase movement of ISF into the airways and increase drug concentrations in this space. Inflammation could also increase the protein concentration and protein binding in the PELF leading to higher concentrations in this matrix. Additionally, the peak concentration in the PELF could have been missed as the concentration steadily decreased after our initial sample at 6 hours, well after the plasma *T*_MAX_. Previous studies showed a later PELF *T*_MAX_ for enrofloxacin in healthy steers,^16^ suggesting that PRA could reach peak concentrations in the airways earlier than enrofloxacin.

For fluoroquinolones, an AUC/MIC >72 for the free fraction of drug (*f*AUC) is the pharmacodynamic target based on recommendations from CLSI and USCAST. The unbound fraction in plasma is used as a surrogate measure of efficacy for pharmacokinetic‐pharmacodynamic (PK/PD) assessments. The mean *f*AUC for pradofloxacin in plasma was 7.25, which produces a *f*AUC/MIC over 480 for the isolate used in this study and is consistent with clinical efficacy as demonstrated here.

In this study, florfenicol PK parameters differed from those in our previous work.^5^ The AUC was less in the plasma (102 vs 143 h × μg/mL), ISF (67 vs 137 h × μg/mL), and PELF (235 vs 342 h × μg/mL) in this study. The penetration into the ISF (66% vs 97%) and PELF (230% vs 260%) was also lower. Because these were different groups of calves with different disease conditions (sick vs healthy) and different studies, we cannot be certain of the cause for these differences. Potentially, experimentally induced BRD increased protein binding of florfenicol, reducing the penetration into the ISF. If we use the ratio of ISF/plasma AUC as a measure of in vivo protein binding, the value from this study is a mean of 0.65 (Table 3) which is lower than previously shown in healthy cattle (95%).^5^ Plasma *f*AUC/MIC is described as the best predictor of clinical efficacy for florfenicol for respiratory pathogens in calves. The PK‐PD target for florfenicol in calves is an AUC/MIC >24.^34^, ^35^ Even including protein binding as high as 35%, based on our calculated penetration factor of 0.65, the *f*AUC/MIC is still well above 24. As with pradofloxacin, this value should be consistent with clinical improvement in the steers, which we observed.

Tulathromycin had significantly lower penetration into the ISF compared with PRA and FLOR, and less than previously demonstrated in healthy steers (21% vs 44%).^5^ As discussed above for florfenicol, it may be possible that protein binding increased during infection because of binding to acute phase proteins, thus limiting penetration into the ISF. The presence of infection in calves did not appear to increase penetration of tulathromycin into transudate or exudate as in other studies.^36^ Penetration into the PELF was significantly higher than PRA, but not FLOR. TUL penetration into the PELF was less than previously demonstrated in healthy steers (519% vs 905%).^5^ Our observed lower penetration in the PELF during pulmonary infection compared to penetration in healthy calves supports the conclusion by others that leukocyte infiltration into inflammatory airways may not be an important source of an antibiotic reservoir in the lungs.^37^ In prior studies, this observation may have been the result of lysis of leukocytes during bronchoalveolar lavage. Because tulathromycin has relatively lower ISF concentrations, movement of ISF into the airways following inflammation may lower the drug concentration because of dilution, explaining the reduction in penetration into the PELF. As tulathromycin is seen as an effective treatment for BRD, the clinical relevance of the differences seen in penetration into the PELF are unclear.

The optimum PK‐PD parameter is unclear, but an AUC24/MIC >24 was determined from time‐kill studies of *Mannheimia* for tulathromycin in calves.^36^ If we assume a protein binding of tulathromycin in calf plasma of 32%‐39%, the fraction unbound in calves is 0.61‐0.68^5^, ^38^ the mean plasma *f*AUC24/MIC in our study was only 1.3 and 2.7 for the MICs reported of 4 and 8 μg/mL, respectively. This is lower than the *f*AUC24/MIC calculated for tulathromycin in a calf infection model^36^; however, the MH strain in their study had a mean MIC of 2.07 μg/mL. The approved FDA label for tulathromycin lists a MIC_90_ of 1.0 μg/mL with a range 0.5‐64 μg/mL. Their study also reported a much higher AUC and longer half‐life than in our present study. Other studies^39^, ^40^ reported values similar to ours, and another study was lower.^41^ The cause of these discordant results is undetermined.

The clinical outcomes for the TUL steers were similar to the PRA and FLOR groups. This treatment result might not be attributable to high concentrations in the PELF as suggested by others^42^ because the PELF concentrations in our study never exceeded the MIC of the bacteria. This could suggest that, like other macrolides,^43^ tulathromycin can exert additional non‐microbiological effects in calves contributing to treatment success.^42^, ^44^, ^45^

The only significantly different clinical finding was an increased percentage of TUL calves with lung consolidation on ultrasound. While previous studies have shown clinical importance of ultrasonographically detected lesions,^22^, ^23^ the clinical relevance of this in light of not seeing significant differences in lung consolidation at necropsy is unclear. Five steers in each group had lung consolidation seen at necropsy, while no steers had visible lung consolidation on ultrasound before euthanasia. Likely, the overlying normally inflated lung prevented detection of lung consolidation by ultrasound. Previous studies of thoracic ultrasonography demonstrate good agreement between ultrasound findings and necropsy lesions in clinical disease.^46^ Yet, these findings illustrate the limits of ultrasound in assessing lung consolidation in an experimental study where consolidation may be more localized and deeper in the lungs. Further, this study was not designed or powered to assess diagnostic accuracy of thoracic ultrasonography.

The infection model used in this study was based on 2 different methods^18^, ^19^ because in a pilot study, nebulization with MH did not reliably induce disease in these older steers. With this combined approach, all steers met the inclusion criteria by 36 hours post challenge. Necropsy demonstrated that we consistently inoculated the right main stem bronchus as all pathologic findings were in the right lung, suggesting that directly instilling MH was the cause of disease, not nebulization.

Both the MH inoculation and sampling of PELF were performed blindly; therefore, we cannot determine if we collected the PELF samples from the diseased area of the lungs. Yet, the procedures were performed in a similar manner, and given the consistency of inoculation of the right diaphragmatic lobe, one would expect that the sampling was also routinely in the right main stem bronchus. Endoscopic sampling could ensure sampling from a consistent location, but this is not possible with swab sampling as the filter paper is too large to fit through an endoscopic sampling port.

Twenty isolates of MH were found after treatment across the 3 groups. The proportion of steers positive after treatment was not different between groups. Based on PFGE, all 20 isolates are the same as the initial isolate, indicating that no treatment cleared the bacteria in all steers. The MIC of all isolates were within 1 dilution of the MIC in the initial isolate, suggesting that mutation to a more resistant MH was not the reason for persistent carriage. The MIC for this isolate was similar to published surveys of MH isolates from cattle. The MIC for PRA (<0.016 μg/mL) was the same as recently published for 34 different clinical isolates.^47^ The MIC for FLOR (0.5 μg/mL) was less than 2 studies (MIC_50_ = 1 μg/mL)^47^, ^48^ and similar to a third.^49^ The MIC for TUL (8 μg/mL) was greater than 2 studies (MIC_50_ = 2 μg/mL)^47^, ^49^ and similar to a third.^48^

Limitations of this study include its relatively small size. The group sizes were based previous PK studies, and therefore, the study was underpowered to demonstrate differences in clinical outcomes, and we may have missed differences that could have been apparent in larger studies. While every attempt was made to recreate clinical disease (transport stress, comingling, exposure to MH), this infection is different than clinical BRD. This is most obvious in distribution of lesions at necropsy. As we did not have an untreated control group, the severity of the challenge model cannot be fully evaluated as clinical signs resolved in all treated steers. This challenge model may not have been severe enough to demonstrate differences between outcomes of the drugs, and this could have also limited our ability to identify lesions via ultrasound. Additionally, clinical outcomes could have been affected by the repeated handling and sampling of steers. Both the FLOR and TUL groups required prolonged sampling relative to the PRA group which could have worsened outcomes for those 2 groups. Another difference in the treatment groups was the inclusion of flunixin in the FLOR as a combination product was used, which could have affected the outcomes that were seen. The age and size of steers was more variable because of study interruptions by the COVID‐19 pandemic. These age and size differences could increase the variability in the infection model as they all received the same inoculum.

We demonstrated that pradofloxacin and florfenicol achieve high PELF concentrations in steers with experimentally‐induced BRD, and exceed plasma PK‐PD targets for the MH isolate used in this study. Tulathromycin concentrations were below the MIC of the MH isolate throughout the study. Penetration of pradofloxacin into the airways appears to peak earlier than enrofloxacin in healthy steers, and penetration of florfenicol and tulathromycin appear to be reduced in animals with BRD compared to studies in healthy cattle. While ultrasonographic lung lesions were more common in tulathromycin treated steers, all other clinical outcomes were not significantly different between the 3 treatment groups.

## CONFLICT OF INTEREST DECLARATION

Drs. Papich and Foster have previously received payment for consulting, speaking, and travel by various pharmaceutical companies including the manufacturers of the drugs in this study, Elanco Animal Health. To minimize the risk of bias, animals were randomly assigned to treatment groups through a random number list, and only 1 member of the research team was aware of the treatment group assignments. This person administered all treatments and was not involved in any clinical scoring or necropsy scoring. No representatives from Elanco Animal Health were involved in performing any part of the research trial or writing any aspect of the manuscript, though they have reviewed it.

## OFF‐LABEL ANTIMICROBIAL DECLARATION

Pradofloxacin was not approved for use in cattle at the time of this study, but was approved in April 9, 2024 by the United States Food and Drug Administration.

## INSTITUTIONAL ANIMAL CARE AND USE COMMITTEE (IACUC) OR OTHER APPROVAL DECLARATION

All animal use was approved by the North Carolina State University IACUC (20‐499).

## HUMAN ETHICS APPROVAL DECLARATION

Authors declare human ethics approval was not needed for this study.
